# Therapeutic Plasma Exchange in Multiple Sclerosis and Autoimmune Encephalitis: A Comparative Study of Indication, Efficacy, and Safety

**DOI:** 10.3390/brainsci9100267

**Published:** 2019-10-09

**Authors:** Tobias Moser, Gayane Harutyunyan, Anush Karamyan, Ferdinand Otto, Carola Bacher, Vaclav Chroust, Markus Leitinger, Helmut F. Novak, Eugen Trinka, Johann Sellner

**Affiliations:** 1 Department of Neurology, Christian Doppler Medical Center, Paracelsus Medical University, 5020 Salzburg, Austria; t.moser@salk.at (T.M.); gayane.harutyunyan@stud.pmu.ac.at (G.H.); anush.karamyan@stud.pmu.ac.at (A.K.); f.otto@salk.at (F.O.); ca.bacher@salk.at (C.B.); info@neurologie-chroust.at (V.C.); ma.leitinger@salk.at (M.L.); h.novak@salk.at (H.F.N.); e.trinka@salk.at (E.T.); 2 Department of Neurology, Klinikum rechts der Isar, Technische Universität München, 81675 München, Germany; 3 Department of Neurology, Landesklinikum Mistelbach-Gänserndorf, 2130 Mistelbach, Austria

**Keywords:** multiple sclerosis, autoimmune encephalitis, plasma exchange, autoimmunity, immunotherapeutics, clinical outcomes

## Abstract

Therapeutic plasma exchange (TPE) is a well-established method of treatment for steroid-refractory relapses in multiple sclerosis (MS) and neuromyelitis optica spectrum disorders (NMOSD). Little is known about indications and clinical responses to TPE in autoimmune encephalitis and other immune-mediated disorders of the central nervous system (CNS). We performed a retrospective chart review of patients with immune-mediated disorders of the CNS undergoing TPE at our tertiary care center between 2003 and 2015. The response to TPE within a 3- to 6-month follow-up was scored with an established rating system. We identified 40 patients including 21 patients with multiple sclerosis (MS, 52.5%), 12 with autoimmune encephalitis (AE, 30%), and 7 with other immune-mediated CNS disorders (17.5%). Among patients with AE, eight patients had definite AE (Immunolobulin G for N-methyl-D-aspartate receptor *n* = 4, Leucine-rich, glioma inactivated 1 *n* = 2, Ma 2 *n* = 1, and Alpha-amino-3-hydroxy-5-methyl-4-isoxazolepropionic Acid *n* = 1). Intravenous immunoglobulins had been given prior to TPE in all but one patient with AE, and indications were dominated by acute psychosis and epileptic seizures. While TPE has a distinct place in the treatment sequence of different immune-mediated CNS disorders, we found consistent efficacy and safety. Further research should be directed toward alternative management strategies in non-responders.

## 1. Introduction

Apheresis therapies separate patients’ plasma from the whole blood by using centrifugation devices or highly permeable filters. In this regard, therapeutic plasma exchange (TPE) aims to eliminate pathogenic antibodies and other proinflammatory mediators from the patient’s circulation. This procedure is an established treatment for steroid-refractory relapses in multiple sclerosis (MS) [1]. Several studies corroborated its efficacy in about 66%–86% of patients undergoing TPE, after conventional high-dose glucocorticoid (GC) treatment had failed [2,3,4]. The rationale for treatment of acute and recurrent attacks in neuromyelitis optica spectrum disorders (NMOSD) is based upon evidence that humoral autoimmunity plays a key role in the pathogenesis. Of note, the interest to achieve rapid remission in NMOSD is driven by the high attack-related disability and -mortality [5]. Therefore, a more aggressive treatment concept based on immunosuppression, pulsed immunotherapy, or targeted disruption of the immunological cascade leading to neuroaxonal injury is maintained in NMOSD in order to preserve long-term neurological function [6].

The spectrum of immune-mediated disorders of the CNS widened over the recent decade. There is emerging evidence that GCs are less effective in B-cell-mediated diseases, including autoimmune encephalitis, and TPE is likely to be effective from a pathophysiological viewpoint in the treatment of antibody-mediated immune processes [7,8]. Autoimmune encephalitis (AE) is a clinical challenge, since presentation is unspecific, and therefore diagnostic consideration is often delayed. Moreover, some patients require treatment at the intensive care unit (ICU), and outcomes can be devastating [9]. Notably, due to the relatively recent discovery of anti-neuronal antibodies and the rarity of AE, treatment recommendations are based on retrospective reports and expert opinion. In a study of 30 patients with AE, 67% improved with TPE by at least 1 point in the modified ranking scale (mRS) [10]. There are, however, two other components of treatment of AE [11]. These include intravenous immunoglobulins (IVIG) and tumor removal. Of note, the ideal sequence for GC, IVIG, and TPE has not been established yet. In addition, there is emerging evidence for the efficacy of rituximab, a CD20 depleting antibody, for achieving long-term remission [12]. Moreover, TPE is not without risks and should only be carried out in conditions where there is good evidence of its effectiveness. Side-effects include disturbances of coagulation, vasovagal episodes, fluid overload or under-replacement, and allergic or anaphylactic reactions due to plasma infusion [13]. Immunoadsorption (IA) is a selective technique for the removal of autoantibodies and immune complexes with less adverse effects in contrast to TPE, which is a non-selective extracorporeal blood purification process with elimination of plasma and subsequent substitution. Recent studies have shown that IA is not only effective in GC-unresponsive MS relapses but also in exacerbations related to NMOSD [14,15].

Here, we hypothesized that TPE is effective in autoimmune encephalitis and therefore studied indication, efficacy, and safety in comparison with MS and other immune-mediated disorders of the CNS.

## 2. Materials and Methods

### 2.1. Study Design

We performed a retrospective chart review of all patients with immune-mediated disorders of the CNS who underwent TPE at the 9-bed neurological intensive care unit (NICU) of a tertiary university hospital (Christian Doppler Medical Center, Paracelsus Medical University, Salzburg, Austria). The study protocol was reviewed and approved by the local Ethics Committee (Ethikkommission für das Bundesland Salzburg; 415-EP/73/534-2015).

### 2.2. Study Population and Data Collection

We reviewed the electronic records for demographic data, neurological diagnosis, symptoms, complications, number of TPE cycles, and outcome and included patients according to the following inclusion criteria:

- acute immune-mediated disorder of the CNS
- TPE during the period of January 2003 to December 2015
- sufficient clinical documentation on underlying disease, indication, procedures, and complications

Multiple sclerosis was diagnosed according to the McDonald’s criteria revised in 2010 [16]. For AE, we followed the diagnostic criteria set up by Graus and coworkers [4]. Briefly, diagnosis can be made when all three of the following criteria have been met:

- subacute onset (rapid progression of less than three months) of working memory deficits (short-term memory loss), altered mental status, or psychiatric symptoms
- at least one of the following: new focal CNS findings, seizures not explained by a previously known seizure disorder, CSF pleocytosis (white blood cell count of more than five cells per μL), MRI features suggestive of encephalitis

**Figure 1:**
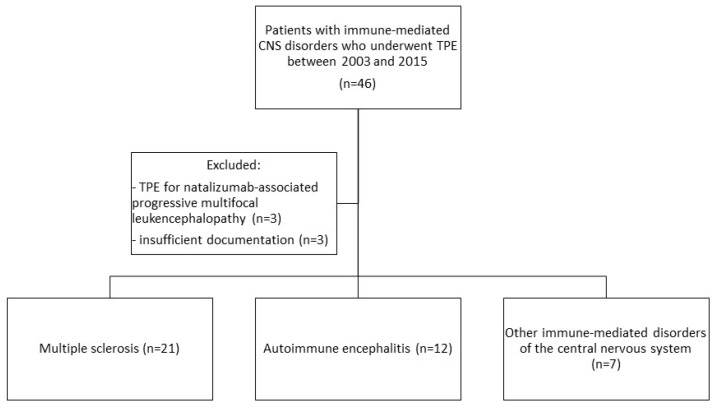
Flow chart for patient selection.

The cohort of patients with “other immune-mediated CNS disorders” comprised acute disseminated encephalomyelitis (ADEM), CNS lupus, optic neuritis not related to MS, and NMOSD. MS patients with progressive multifocal leukoencephalopathy (PML, *n* = 3) who received TPE for elimination of natalizumab were excluded. Three patients with AE were excluded for lack of sufficient follow-up (Figure 1).

TPE was performed by experienced neurointensivists via a central venous access catheter. The clinical response to TPE was rated with a scoring system introduced by Magaña et al. [6,17,18], which proposes three different response categories; these were no, mild, and good improvement. Patients in the first group showed no recovery at all or even deterioration of symptoms. Mild recovery was defined as “improvement in neurological status without impacting function”. Individuals with functionally relevant neurological recovery were considered to have a good improvement. We studied the outcome within six months from TPE. If patients were not seen regularly on follow-up at our center, we contacted individual patients or their caregivers to collect information on their recovery.

### 2.3. Statistical Analysis

All statistical analyses were conducted using IBM SPSS Version 21.0 (SPSS, Chicago, IL, USA). Descriptive statistics for clinical, demographic, and outcome data are provided. We report the median (interquartile range, IQR) for continuous variables and frequency (percent) for categorical variables. Intergroup comparisons were performed using Fisher’s exact and Mann–Whitney U tests and one-way ANOVA where appropriate. All reported p-values were two-tailed and considered statistically significant at *p* < 0.05.

## 3. Results

### 3.1. Clinical and Demographic Characteristics

We identified a total of 40 patients with immune-mediated CNS disorders who underwent PLEX between 2003 and 2015. As shown in Figure 1, the most frequent condition was MS in 52.5% (*n* = 21), followed by AE in 30% (*n* = 12). The remaining disorders (summarized as “others” in the following) were CNS lupus (*n* = 3), optic neuritis (not otherwise specified, *n* = 2), ADEM (*n* = 1), and NMOSD (*n* = 1).

**Figure 2:**
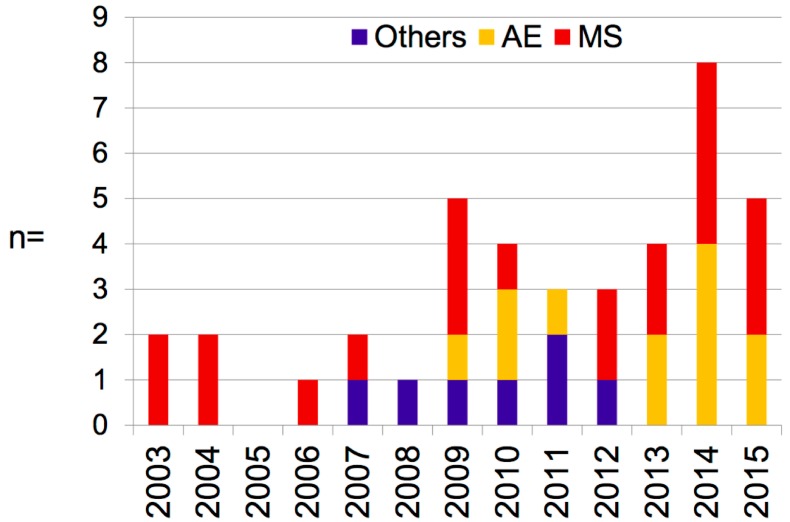
Time course of therapeutic plasma exchange (TPE) usage during the observation period.

There was a general trend towards the increased utilization of TPE over time (Figure 2). Half (*n* = 20) of all patients received the treatment within the past four years, whereas in the period of 2003–2008, only eight patients were treated with TPE.

The majority of patients were women (*n* = 26, 65%). In detail, women were more frequent in the MS group (*n* = 15, 71%), and the group of “other” immune-mediated CNS disorders comprised entirely women (100%). Of note, there were more male patients in the AE group (*n* = 27, 67%).

The mean age of patients with MS was 35.5 (standard deviation (SD) of 9.4). TPE was used in this group as second-line therapy except in one patient where steroids were contraindicated, as shown in Table 1. The most frequent indication was optic neuritis (*n* = 9), followed by pyramidal tract symptoms (*n* = 6). The mean number of TPE courses was 5.1 (range of 2–9). There were single patients with eight and nine TPE cycles, respectively.

The mean age of patients with AE was 45.1 years (SD of 18.8), which was significantly higher than in MS (*p* = 0.03). The spectrum of clinical symptoms in patients with AE was broad, and the most frequent disturbances were psychiatric symptoms and epileptic seizures (Table 2). The average number of TPE cycles in patients with AE was 6.3 (SD of 2.7). Two patients had more than 10 cycles of TPE. In almost all patients, a prior treatment with intravenous immunoglobulins was performed. Some also received steroids prior to TPE. In the AE cohort (*n* = 12), eight (66%) patients had definite AE with positive antibodies (IgG for NMDA-R: *n* = 4, LGI1: *n* = 2, Ma 2: *n* = 1, and AMPA: *n* = 1). Six patients had inflammatory CSF and eight had pathologies on MRI. All except the patients with exclusively brainstem involvement developed neurocognitive signs, and seizures were common (60%). The four patients with anti-NMDAR-antibodies were women, two of whom had a histologically confirmed teratoma. All of the AE patients had additional treatments (surgery *n* = 4, steroids *n* = 5, IVIG *n* = 11, rituximab *n* = 2).

The mean age of patients with other autoimmune CNS disorders was 50.7 years (SD 18.1). The mean number of TPE cycles was 5.7 (SD 2.1). Further details are presented in Table 3.

### 3.2. Time from Symptom Onset to Start of TPE

The time from relapse onset to the initiation of TPE was distinct among the three groups (*p* = 0.03). In detail, we calculated the median of 15 days (interquartile range (IQR) 10–27 days) for patients with MS, 77 days (24–203) for patients with AE, and 11 days (9–55) for patients with other immune-mediated disorders of the CNS.

### 3.3. Outcome

**Figure 3:**
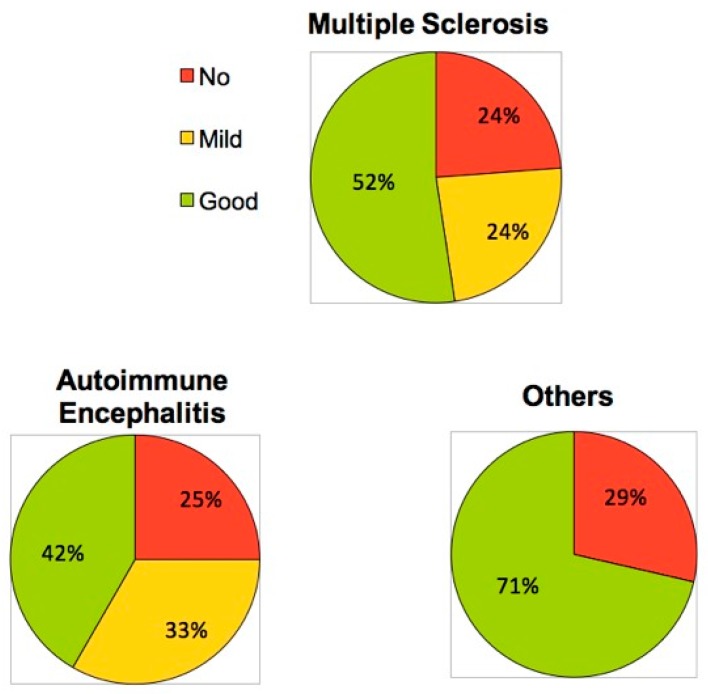
Clinical response to TPE across three subgroups of immune-mediated CNS disorders.

The overall rate of TPE responders was 75%. Further details are shown in Figure 3. A good or mild response was observed in 52.5% and 22.5% of the patients, respectively. The best outcome was observed within the “other CNS-ID” group, where 71% (*n* = 5) had a good recovery after PLEX, while only two patients showed no response (Table 3). The analysis of the MS and AE cohorts disclosed a functional improvement after plasma exchange treatment in 52% and 42%, respectively.

We also evaluated whether the period of symptom onset to the start of the TPE was distinct in patients with good response vs. mild/no response. In a pooled analysis of all three groups we did not find statistical differences (median of 16 days for the cohort with good response vs. 22 days for the cohort with mild or poor response).

### 3.4. Complications

In total, 219 TPEs were performed. Serious complications were found (1.8%): thrombosis of the subclavian vein, episode of bradycardia with transient loss of consciousness (one each), and two patients had impaired coagulation. No cases of death were reported.

## 4. Discussion

This study demonstrates that the majority of patients suffering from immune-mediated CNS disorders benefit from TPE, and the rate of non-responders is similar throughout the conditions. Moreover, the number of patients with AE treated with TPE is increasing over time, which reflects increased recognition of TPE as an effective treatment option. Of note, 50% of our patients with AE received TPE for intractable seizures and status epilepticus. Recent studies indicate that immune-mediated epilepsy such as in AE responds better to immunotherapies than to conventional epilepsy therapies [19]. Complications of TPE in our patient series was in the range of previously published studies in the real-life setting [3,20,21].

In our study, the rate of good recovery was higher in MS patients than in AE. This is could be related to the more complex pathogenesis and requirement of several lines of treatments for effective treatment of AE [10,22,23,24]. Thus, we cannot exclude the overlapping effects of prior treatment approaches. Among the patients with AE and no recovery after TPE, one suffered from a typical paraneoplastic syndrome with intracellular antibodies (MA1/2), which is to date believed to show no or limited response to TPE. In another patient with AE and poor response, the follow-up may have been too short. According to a study by Titulaer et al., almost half of the patients with NMDAR-encephalitis need a prolonged immune-suppressive treatment, and clinical improvement can be delayed [10]. Therefore, a delayed recovery cannot be excluded. Indeed, comparing two disorders with different pathogenesis is problematic. While AE may be monophasic, MS is mostly a relapsing-remitting disease. A B-cell-mediated pathogenesis is implicated in both AE and other CNS IDs, which backs the use of TPE, whereas this is the predominant process of acute inflammation in only a subset of MS patients [7,25]. Most importantly, time to TPE seems to be one of the most critical factors for observing an adequate treatment response [10,22,24]. Neurological involvement is relatively common in the majority of systemic autoimmune diseases and may lead to severe morbidity and mortality, if not treated promptly [26,27]. While our findings further support the use of TPE in neurological complications of systemic disease, our literature search revealed only a few case series for this indication [28,29]. Thus, prospective studies and establishment of registries are eagerly awaited. Open questions include the number of TPE cycles, definition of treatment goals and development of biomarkers. In addition, further studies should also elucidate the role of immunoadsorption as an alternative treatment option.

Moreover, the exact mechanism of action of TPE it is still incompletely understood. The removal of immunoglobulins seems to be a crucial factor. This process is likely to be followed by a shift from cerebral tissue towards systemic circulation [11]. TPE also modulates the immune system by changing the lymphocyte distribution, including changes in B and T cell numbers and activation, increased T suppressor function, and alteration in T-helper cell type 1/2 (Th1/Th2) ratio. In contrast, steroids alone often insufficiently resolve autoantibody-mediated pathologies [13]. A small retrospective review of 14 patients with NMDAR-encephalitis reports better outcomes in patients receiving TPE shortly after GCs than those with GC-treatment alone [12]. In our cohort, none of the 12 patients with AE were treated with TPE alone in the acute disease phase: 10 (83%) had additional IVIG and 6 (50%) had steroid treatment. Because adverse events are estimated to occur in about 6% of every single TPE procedure (the risk for the single individual is therefore multiplied by the number of exchanges), it was used as the ultima ratio treatment after failure of IVIG and/or GCs. Ehlers et al. studied a cohort of 37 GCS-unresponsive MS patients and reported a median time from symptom onset to begin of TPE of 44 days (range of 11–154) [30]. The median time in our MS cohort was 15 days, whereas 77 days had elapsed in patients with AE since the begin of clinical symptoms. This interval was even shorter for patient with other ID of the CNS (median 11 days). In the latter group were patients with known pathogenetic role of B-cells and humoral immunity including NMOSD and neurological manifestations of lupus. Moreover, due to this time lag, a delayed response to immunotherapies given prior to TPE is less likely. The long interval in AE is noteworthy and we assume that the approach for treatment escalation with TPE has changed over the recent years with knowledge about the key importance of an early TPE use in case of GCS-unresponsiveness and implementation in treatment guidelines [31]. In this regard, early initiation of TPE and lower patient age have been reported as predictors of a good response [22]. It also should be noted that none of our patients were treated with IA, which is an emerging treatment option in MS and CNS ID [2,15,32].

We report a low rate of adverse reactions. It should be noted that we exclusively used a central venous access which carries a significantly higher risk of complications than TPE via a peripheral line. In a recent study of complicated SLE and other autoimmune conditions (*n* = 66) using a central access device, the majority of complications were mild, with bleeding (25.8%) being the most common [28]. Electrolyte disturbances, hypotension, mild arrhythmia, and hypersensitivity were reported occasionally (6.1%, 9.1%, 3%, and 1.5%, respectively). Notably, 27.3% developed infections in the 14 days after TPE.

Improvement in function was achieved in 52.5% of all patients, which is in concert with TPE response rates found in literature ranging from 40% to 63%. Further subgroup analysis including analysis of predictors, however, could not be made due to the limited size of our cohort. Another limitation in this study is the retrospective character and the lack of a control-group.

## 5. Conclusions

Taken together we conclude that TPE shows similar response rates throughout different CNS IDs. Further studies should focus on the prediction of non-responders and development of alternative treatment strategies for these patients.

## Figures and Tables

**Table 1:** TPE in patients with multiple sclerosis.

No.	Age	Gender	Indication	GC Refractory	Courses of TPE	Clinical Response
1	42	M	GC contraindicated		5	good
2	37	F	optic neuritis	X	5	good
3	39	F	optic neuritis	X	5	good
4	44	F	optic neuritis	X	5	good
5	25	F	optic neuritis	X	5	mild
6	40	F	optic neuritis	X	5	mild
7	28	F	optic neuritis	X	5	mild
8	28	M	optic neuritis	X	5	mild
9	47	M	optic neuritis	X	5	no
10	33	F	optic neuritis	X	5	no
11	43	F	tetraparesis	X	2	good
12	23	M	tetraplegia	X	5	good
13	33	M	hemiparesis, dysarthria	X	5	good
14	17	M	hemiparesis	X	5	good
15	34	F	tetraparesis	X	5	mild
16	44	F	tetraplegia	X	8	no
17	50	M	tetrapresis, dysarthia, dysphagia	X	5	no
18	43	F	hemiparesis, ataxia	X	3	no
19	25	F	fulminant MRI, aphasia	X	5	good
20	29	F	fulminant MRI, natalizumab rebound	X	5	good
21	21	F	fulminant radiological findings	X	9	good

Legends: M, male; F, Female; MRI, magnetic resonance imaging; GC, glucocorticoid.

**Table 2:** TPE in patients with autoimmune encephalitis (AE).

n	Age	Gender	Details	Antibody	Memo	Epil	Psych	Move	Auto	Sleep	Pons	Detection of Lesion on MRI	CSF	Treatment	TPE Courses	Clinical Response
1	42	M	Paraneopl.AE	Ma1/Ma2	+	+	+			+		temporomesial	neg.	TPE/OP/Ritux	3	no
2	29	W	NMDAR-E	NMDAR		+	+	+				diffuse	IgG ↑	TPE/IVIG	10	good
3	26	W	NMDAR-E	NMDAR			+		+			none	33 cells	TPE/IVIG/OP	5	good
4	30	W	NMDAR-E	NMDAR		+	+					none	neg.	TPE/IVIG/OP	5	good
5	25	F	NMDAR-E	NMDAR		+	+	+				none	10 cells	TPE/IVIG/GC/OP	13	no
6	64	M	LE	LGI-1/VGKC	+	+	+					temporomesial	9 cells	TPE/IVIG/GC	5	mild
7	55	M	LE	none	+							temporomesial	21 cells	TPE/IVIG	5	mild
8	62	M	LE	AMPA-R1	+		+					temporomesial	16 cells	TPE/IVIG/Ritux	7	no
9	66	M	LE	LGI-1			+	+				none	neg.	TPE/ IVIG/ GC	4	good
10	24	F	probable LE	none		+	+			+		temperomesial	neg.	TPE/IVIG/GC	5	mild
11	71	M	Brainstem E	none							+	pons	neg.	TPE/IVIG	7	good
12	47	M	Brainstem E	none							+	pons	neg.	TPE/IVIG/GC	6	mild

Legends: LE: Limbic Encephalitis; NMDAR-E: NMDAR-Encephalitis; Brainstem E: Brainstem Encephalitis; memo: memory-deficit; epi: epileptic seizure; psych: psychiatric disorders; move: movement disorders; auto: autonomic dysfunction; sleep: sleep disturbance; pons: pontine signs; IVIG: intravenous immunoglobulins; GC: Glucocorticoids; OP: tumor surgery; Ritux: Rituximab.

**Table 3:** TPE in patients with other immune-mediated disorders of the central nervous system (CNS).

No.	Age	Gender	Condition	TPE Courses (*n*)	Outcome
**1**	60	W	CNS-lupus	4	no
**2**	55	W	CNS-lupus	4	good
**3**	59	W	CNS-lupus	7	good
**4**	27	W	optic neuritis	5	good
**5**	47	W	optic neuritis	10	mild
**6**	78	W	NMOSD	5	no
**7**	29	W	ADEM	5	good

## Abbreviations

ADEM	Acute disseminated encephalomyelitis
AE	Autoimmune encephalitis
CNS-ID	Central nervous system-inflammatory disorders
GC	Glucocorticosteroids
IVIG	Intravenous immunoglobulins
LE	Limbic encephalitis
MS	Multiple sclerosis
NMDAR	N-methyl-D-aspartate-receptor encephalitis
NMOSD	Neuromyelitis optica spectrum disorder
NICU	Neurological intensive care unit
ON	Optic neuritis
TPE	Therapeutic plasma exchange
